# Successful treatment of a complete Susac syndrome in a 16‐year‐old boy: A case report

**DOI:** 10.1002/ccr3.9027

**Published:** 2024-07-24

**Authors:** Davood Kashipazha, Mohammad Ali Bahramy, Mahshad Razaghi, Zeinab Rahimi

**Affiliations:** ^1^ Department of Neurology Ahvaz Jundishapur University of Medical Sciences Ahvaz Iran; ^2^ Department of Neurology Naft Grand Hospital Ahvaz Iran; ^3^ Student Research Committee Shiraz University of Medical Sciences Shiraz Iran

**Keywords:** fluorescein angiography, imaging, pediatric, retinal branch artery occlusion, Susac syndrome, treatment, triad

## Abstract

**Key Clinical Message:**

We reported a pediatric case of SuS with a complete diagnosis triad. Although the optimal treatment of SS is unclear, prompt diagnosis and treatment can result in almost a complete recovery.

**Abstract:**

Susac's syndrome (SuS) is a rare, autoimmune disorder known as a typical triad of sensorineural hearing impairment, central nervous system involvement, and multiple branch retinal artery occlusions (BRAOs). It is usually misdiagnosed or underdiagnosed because its symptoms may vary at the presentation time. Diagnosis can be established based on neuroimaging, ophthalmic examination, and audiometry, which match the clinical symptoms. SuS is very limited and rare in childhood and can be easily misdiagnosed with multiple sclerosis or acute disseminated encephalomyelitis. We report a 16‐year‐old boy patient with a completed SuS triad including BRAO in fluorescent angiography (FA), mild to moderate sensory neural hearing loss (SNHL), “Snowball lesions,” and “pearl of string” signs in magnetic resonance imaging (MRI). Successful treatment was achieved with methylprednisolone, rituximab, azathioprine, cyclophosphamide, and plasmapheresis. SuS is a rare disorder, which rarely presents with a full triad and all the manifestations may not be present at the onset of the disease, leading to misdiagnosis or underdiagnosis. Our case is exceptional because he was in a pediatric age and presented with a complete triad of SuS which adds to the rarity of this disease. Although optimal treatment of SuS is unclear, our treatment regimen resulted in almost a complete recovery.

## 1 INTRODUCTION

Susac's syndrome (SuS) is an infrequent disorder known as a typical triad of sensorineural hearing impairment, encephalopathy, and multiple branch retinal artery occlusions (BRAO) caused by an autoimmune response toward endothelial cells that leads to micro‐infarcts of the retinal area, inner ear, and brain.^1^, ^2^, ^3^, ^4^, ^5^ A high index of suspicion is required for a definite diagnosis while the full triad of SuS is rarely seen at first presentation, which leads to a challenging diagnosis.^6^ SuS is very rare among children and may be misdiagnosed with multiple sclerosis (MS) or acute disseminated encephalomyelitis (ADEM) because of the similar findings.^7^ Herein, we present a pediatric male SuS patient.

## 2 CASE PRESENTATION

A 16‐year‐old middle Eastern male without any significant comorbid family history was admitted due to ataxia, progressive dizziness, headache, confusion, daily fatigue, occasional nausea without vomiting, speech disturbance, and left hemiparesis from 1 week prior to his admission. He was lethargic but he could obey commands and respond to verbal stimuli. Cranial nerve examinations were normal but cerebellar examinations could not be performed. Decreased muscle power of the left extremities to 3/5 and the presence of Babinski reflex on the left side were also documented. Blood serology analysis was unremarkable, with a serum albumin 3.6 g/dL and IgG of 8.05 g/L. The initial cerebrospinal fluid (CSF) analysis showed a negative PCR for HIV, HSV1.2‐CMV, negative ADA‐VDRL, smear culture, IGF index of 7.73, IGG synthesis rate of 60.46, albumin of 7.82, and IGG of 135.20 g/L. Magnetic resonance imaging (MRI) without GAD demonstrated multiple high signal intensity on T2‐weighted and flair sequence in the periventricular white matter, bilateral internal capsule and thalamus, left cerebellar hemisphere and peduncle, body, and splenium of the corpus callosum.

The patient was administered methylprednisolone along with five sessions plasmapheresis with a preliminary impression of ADEM and SuS. The patient was discharged with prednisolone 50 mg/day after relative improvement in symptoms (decreased drowsiness, limited verbal communication, and the ability to sit with help). However, he referred to our center after 2 weeks from his previous admission with a chief complaint of dizziness, headache, drowsiness, ataxia, and left hemiparesis. Based on neurological evaluations, left hemiparesis along with absence of eye contact, speech disturbance, visual impairment in eyes assessed with the Snellen chart (2/10), inability to perform cerebellar examination, bilateral hearing loss, decreased muscle power of the left extremities to 3/5, and brisk deep tendon reflex was reported. The Babinski reflex was present on the left side, and the patient could not sit and stand. The patient's vital signs were stable with no evidence of fever.

**FIGURE 1:**
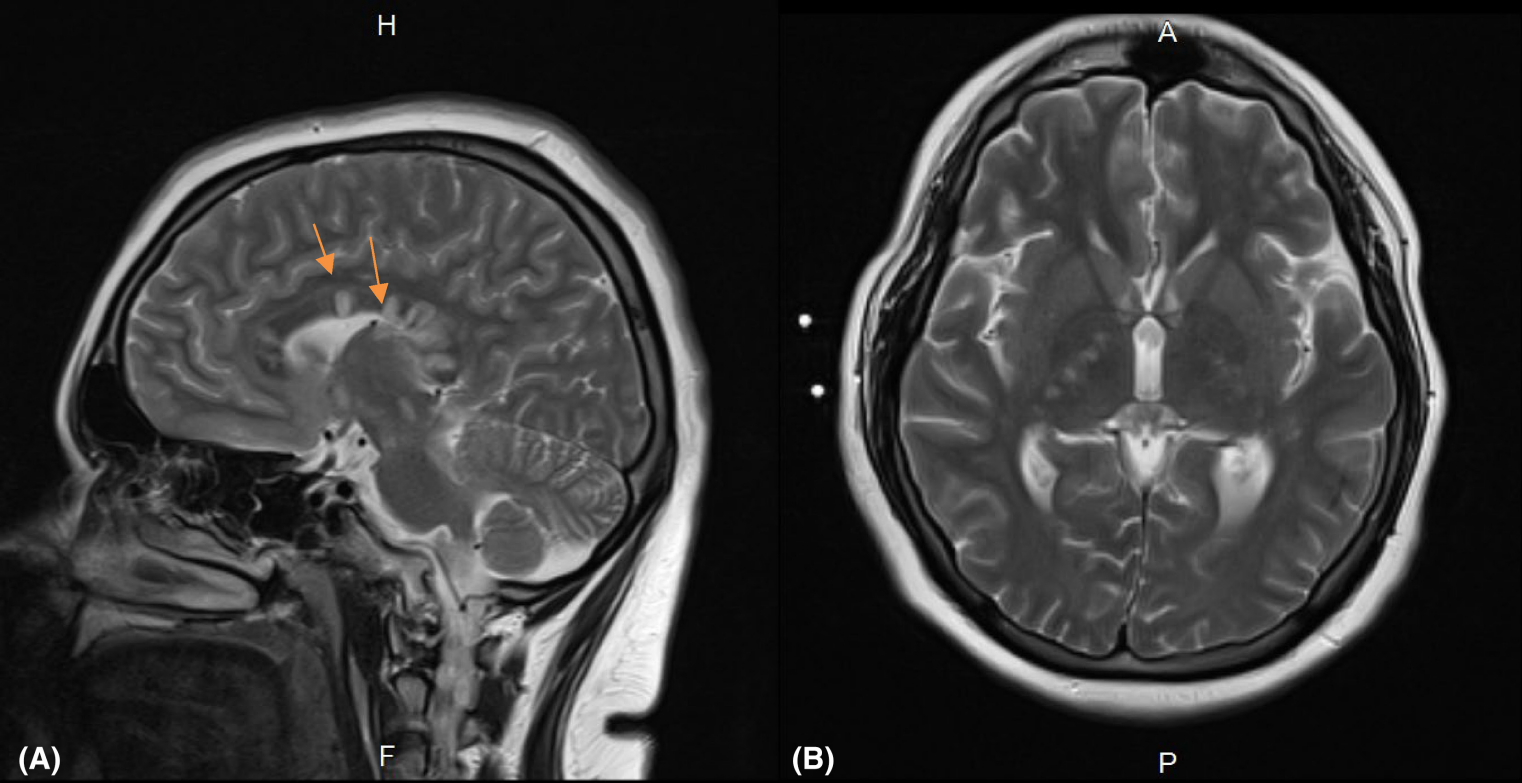
The patient's brain MRI demonstrating (A) hyper‐intense areas on T2‐weighted (yellow arrow), corpus callosum, there was evidence of snowballs, and periventricular and bilateral internal capsule, suggesting a string of pearls (B).

Laboratory evaluations, including routine and vasculitis laboratory analysis, were normal. CSF examination revealed 134 mg of protein and five lymphocytes, and no oligoclonal bands (OCB), which was similar to his previous admission CSF results, which showed total cell count = 8000, red blood cells = 7985, white blood cells = 15, polymorphonuclear neutrophils = 9, lymphocyte = 6, glucose = 60, protein = 130, OCB = negative. Neuroimaging was also similar to his previous MRI, which alongside Diffusion‐weighted images and apparent diffusion coefficient map, demonstrated hyper‐intense areas on T2‐weighted and fluid attenuated inversion recovery sequences (Flair) in the left cerebellar hemisphere, corpus callosum, there was evidence of snowballs, and periventricular and bilateral internal capsule, suggesting a string of pearls (Figure 1). The cervical MRI was normal. There was also no evidence of enhancement with gadolinium.

**FIGURE 2:**
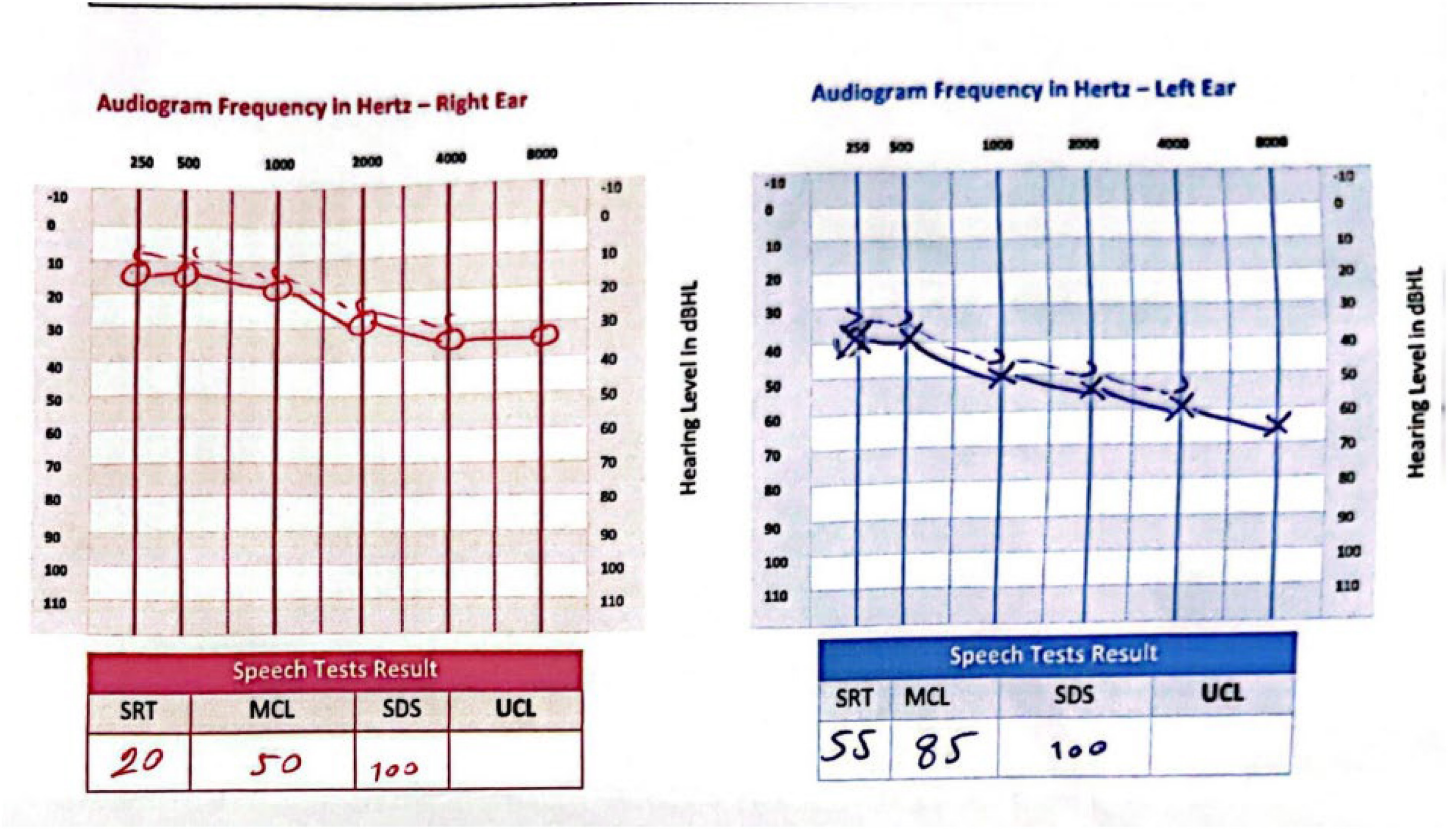
The patient's audiometry report demonstrates mild sensory neural hearing loss in the right ear and moderate to severe sensory neural hearing loss in the left eye.

Audiometry documented mild sensory neural hearing loss (SNHL) in the right ear and moderate to severe SNHL in the left ear (Figure 2).

**FIGURE 3:**
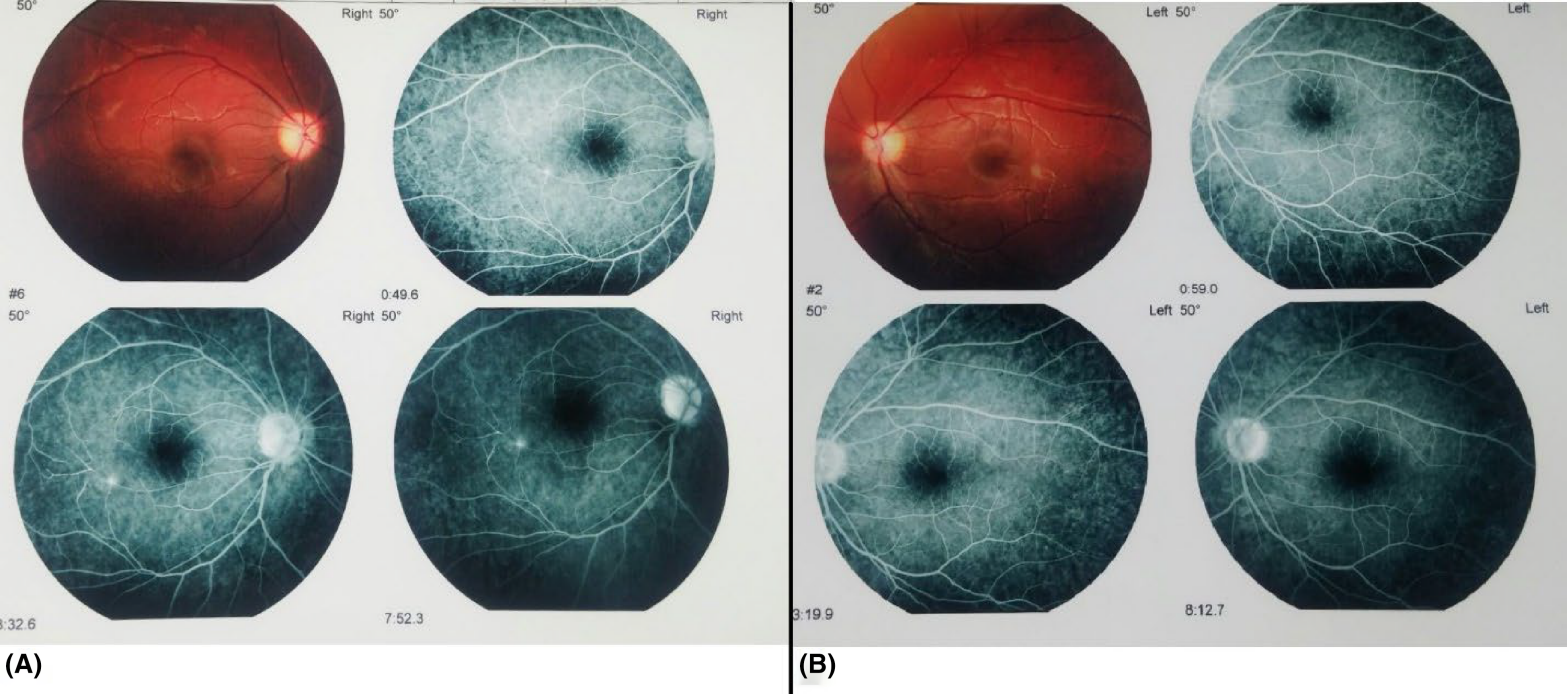
The patient's FA reports BRAO in the left (A) and right (B) eye.

There was evidence of bilateral BRAO in the fluorescein retinal angiography (FA) performed about 5 months after the first episode of the symptoms (Figure 3).

**FIGURE 4:**
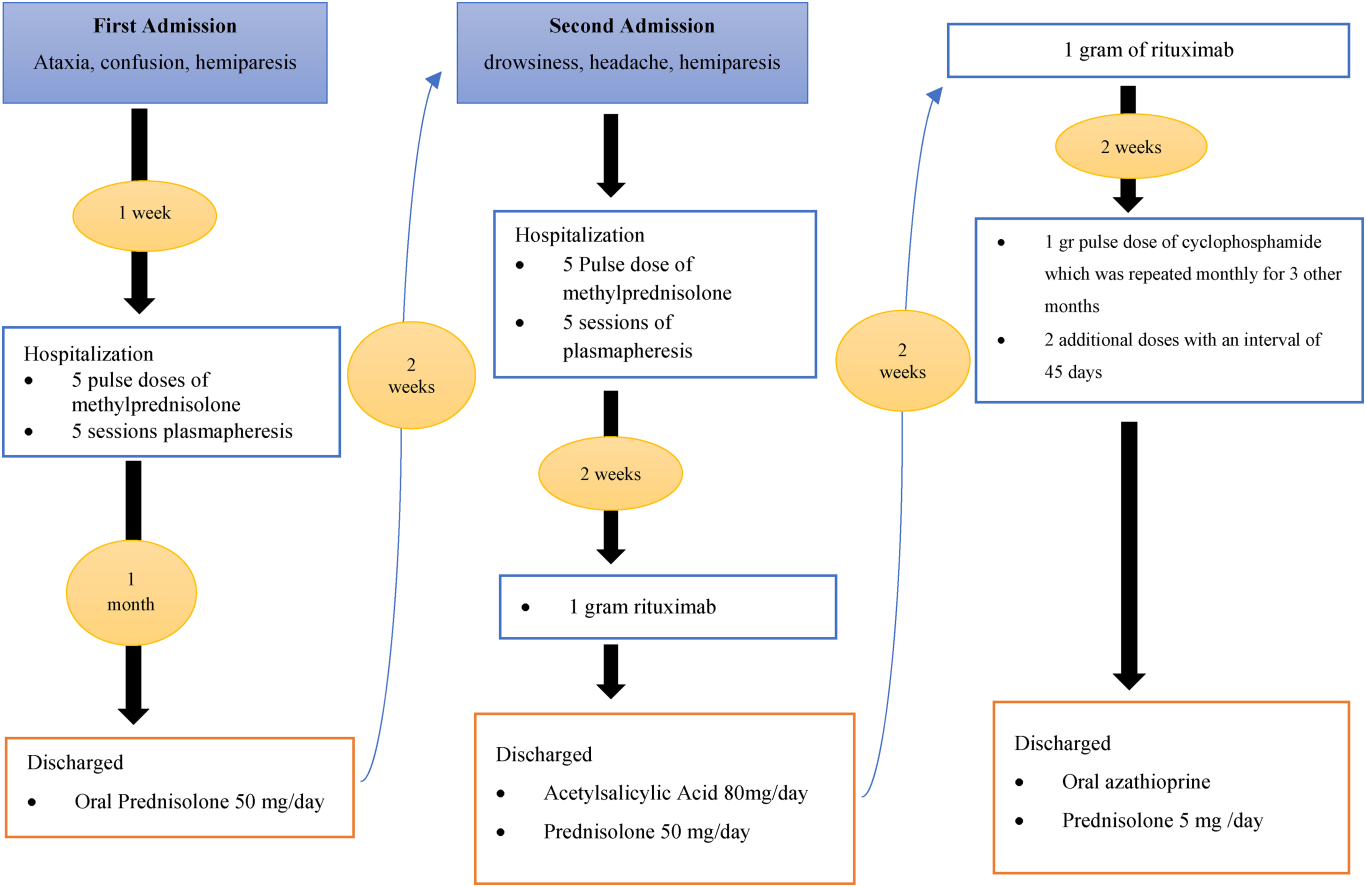
Timeline of events and treatment of a 16‐year‐old boy with Susac syndrome.

Considering the clinical, neuroimaging, and laboratory findings, the diagnosis of complete SuS was made. The results of FA, audiometry, and MRI completely fulfilled the SuS criteria. 1 g Pulse dose of methylprednisolone was administered for 5 days along with 5 sessions plasmapheresis. After 2 weeks, due to lack of improvement in the patients' symptoms, 1 g of intravenous (IV) rituximab was started twice at an interval of 2 weeks. After 2 weeks, due to an incomplete recovery after administrating rituximab, a 1 g pulse dose of cyclophosphamide (endoxan) was started monthly for 4 months followed by two additional doses at an interval of 45 days. He was also started on acetylsalicylic acid (ASA) 80 mg daily and azathioprine. Oral prednisolone was also administered which was gradually tapered to 5 mg after starting first dose of cyclophosphamide. During hospitalization, the patient experienced unilateral facial myoclonic jerks, which was treated with carbamazepine. During his routine follow‐ups, he was able to sit without help after 4 months, walk with the help of others and talk after 6 months, walk alone after 7 months, and run after 10 months after his hospital discharge. Figure 4 illustrates the timeline of events and treatment in our patient.

## 3 DISCUSSION

We report a young male patient, while SuS has been predominantly reported among young women, with a three to one ratio compared. Although the age range of SuS is documented between 7 and 70, pediatric cases of SuS are rare and limited.^7^, ^8^ However, the overall incidence and prevalence are unclear due to the rarity of this disease.^8^ Our case is exceptional as it is a pediatric case with full clinical presentation at the onset of the disease. Although the typical and classic triad, including SNHL, brain, and retinal damages, are pathognomonic for SuS, all clinical presentations might be absent symptomatically or be hard to detect, leading to underdiagnoses or misdiagnosis.^9^ SuS has numerous differential diagnoses including common and infrequent neuro‐inflammatory diseases, notably MS, systemic lupus erythematosus (SLE), ADEM, migraine, Meniere's disease, viral and autoimmune encephalitis and cerebrovascular diseases like cerebral vasculitis.^6^, ^10^ Children with encephalopathy and lesions in the brain white matter might be misdiagnosed as ADEM. However, in our case the presence of sensorineural hearing impairment and BRAOs with corpus callosum involvement is in favor of SuS. Same as adults, MS, Bechet's disease, SLE, sarcoidosis, and tuberculosis may be in the list of differential diagnoses.^7^ A review study by Dorr et al. documented that just 13% of patients have full triad at the beginning of the disease.^8^ Therefore, we should keep in mind that mainly it presents as incomplete SuS and that makes it harder to establish a diagnosis.

Our patient presented with drowsiness, dizziness, headache, and left hemiparesis was present at admission. He was unable to perform a cerebellar examination and verbal communication, and eye contact was impaired which all suggest brain involvement and encephalopathy, which are one of the diagnostic criteria of SuS. As reported earlier, encephalopathy is an initial manifestation of SuS. Patients' symptoms and presentations may vary, including cognitive impairment, psychosis, ataxia, dysarthria, emotional disturbances, and paresis. Migraine‐like headache is the non‐specific and most common symptom affecting about 80% of patients.^8^ So, it is crucial to consider SuS as a differential diagnosis of a variety of diseases in the presence of encephalopathy.

As reported in our patient's MRI on T2‐weighted and FLAIR sequences in the left cerebellar hemisphere, corpus callosum, there was evidence of snowballs, and periventricular and bilateral internal capsule, suggesting a string of pearls. There was no evidence of enhancement with gadolinium. It is in line with the MRI changes reported by other studies supporting that involvement of the brain white matter, deep gray matter, and leptomeningeal enhancement as the classical neuroimaging triad of SuS.^11^ Snowball lesions in the corpus callosum on MRI demonstrate multiple micro‐infarction and are pathognomonic.^12^ There is also another sign on MRI, including “a string of pearls” sign which represents internal capsule micro‐infarction.^13^ Therefore, specific characters shown on MRI can confirm SuS and amplify the importance of radiological evaluation in presenting patients.

CSF examination showed 134 mg protein, five lymphocytes and no OCB were reported. As indicated earlier, elevated protein level and minimal pleocytosis can be seen in CSF.^8^ Although carrying low specificity, CSF analysis is among the routine procedures performed in patients with neurological complaints and can assist in limiting the differential diagnosis and ruling out infectious causes.

In our case, decreased visual acuity evaluated with the Snellen chart (2/10) was reported at the time of presentations, too. As documented before, ophthalmic manifestations include a visual acuity decrease, scotomas, and visual field defects.^8^, ^14^ There was evidence of bilateral BRAO and central retina involvement in his FA. BRAOs seen in FA are the typical retinal presentation in SuS. Patients might have no symptoms if the BRAOs are located in the periphery of the retina. FA has a crucial role in diagnosing of SuS by demonstrating hyper fluorescein of the involved retinal arteriolar walls. Aside from BRAO, Gass plaques, which include yellowish plaques in the retinal arterial wall caused by extravasation of blood and lipids, are frequently seen in SuS patients, but are not specific or pathognomonic for SuS.^14^, ^15^ Latest diagnostic tools, including optical coherence tomography angiography (OCTA) and wide‐field color retinal photography, can be beneficial in earlier diagnosis.^16^ It is crucial for ophthalmologists to carefully know this clinical trio and assess patients with BRAOs, particularly when there are no cardiovascular risks, as it allows early and proper treatment.

On our case's audiometry, it is documented that he had mild SNHL in his right ear and moderate to severe SNHL in his left ear, which can fulfill the triad of SuS. This was consistent with the evidence that the principal characteristic of auditory involvement is low‐frequency SNHL, which may be irreversible, progressive, bilateral, and permanent.^17^

Diagnosis of SuS can be established when there are findings and documentation from retinal involvement shown in FA, MRI, and SNHL in audiometry that matches clinical presentations.^2^ Early identification helps to begin prompt treatment and avoid irreversible serious damages.^18^ When diagnosis and therapy are delayed, irreversible damage related to vision, hearing, and brain is higher. Rapid diagnosis leads to almost complete recovery.^19^

Our treatment regimen consisted of initial pulse dose of methylprednisolone followed by oral prednisolone, rituximab, cyclophosphamide, and azathioprine, which demonstrated a good outcome. SuS is a rare disease for which there is no consistent treatment guidelines, and the treatment regimen should be tailored to the need of the individual patient.^20^ Although the optimal treatment of SS is unclear because of the low number of patients and their different manifestations,^9^ It is recommended to use intensive immunosuppressive therapy, and it is crucial to administer immediate, intensive, and prolonged immunosuppressive treatment when SuS syndrome manifests as encephalopathy.^21^ Empirical treatment including high‐dose corticosteroids is recommended as initial treatment of SuS.^22^ The use of corticosteroids is an appropriate treatment plan for SuS because the disease is caused by inflammatory processes affecting the brain, retina, and ears.^10^, ^22^ There are other medications, such as azathioprine, mycophenolate mofetil, cyclophosphamide, and rituximab, that are recommended for autoimmune diseases.^23^ IV immunoglobulin is also the backbone of treatment when used simultaneously with corticosteroids.^9^ In our patient, due to the encephalopathy and radiological involvement, relative recovery was achieved after administration of IV rituximab, which was improved after receiving cyclophosphamide. There is no obvious indication for immunosuppressive treatment, except for severe patients, for whom other medications, including rituximab and cyclophosphamide, should be given besides glucocorticosteroids. The immunosuppressive treatment plan is based on the principal clinical symptoms, disease severity in individual patients, the possibility of relapse and response to therapy.^19^, ^20^ Thus, combination therapy in SuS may further lead to a positive outcome.

Plasmapheresis was performed for our case and was accompanied by a positive outcome compatible with the findings reported from other studies. The plasma exchange has also been implemented as an ancillary therapy in patients resistant to corticosteroids.^22^ It can be used with other treatments such as corticosteroids and rituximab.^22^ In one case report, a patient with incomplete SuS was treated with methylprednisolone, plasmapheresis, and rituximab, and sustained improvement occurred.^22^ Also, ASA 80 mg was started for him. Adjuvant therapy, including anti‐platelet and anticoagulants, has limited benefits.^19^ So, ancillary treatment besides the main therapy can lead to a better outcome.

## 4 CONCLUSION

Susac's syndrome is an infrequent and rare disorder known as typical manifestations, including sensorineural hearing impairment, encephalopathy, and involvement of the retina due to an immune‐mediated endotheliopathy. All the manifestations may not be present at the onset of the disease, leading to misdiagnosis or underdiagnosis. In cases of clinical suspicion, diagnosis can be established based on neuroimaging such as MRI, ophthalmic examination (FA, OCTA, wide‐field color retinal photography), and audiometry. In literature, pediatric cases are documented limited, but in this study, we draw attention to the happening of this infrequent disease in pediatrics which adds to the rarity of SuS. Early diagnosis is very important because by starting proper treatment early, irreversible damages be can prevented. As mentioned above, optical treatment of SuS is unclear; however, our treatment regimen resulted in satisfactory recovery.

## AUTHOR CONTRIBUTIONS

**Davood Kashipazha:** Conceptualization; supervision. **Mohammad Ali Bahramy:** Conceptualization; data curation. **Mahshad Razaghi:** Data curation; writing – original draft. **Zeinab Rahimi:** Data curation.

## FUNDING INFORMATION

No financial support was received for this case report.

## CONFLICT OF INTEREST STATEMENT

The authors declare that they have no competing interests.

## ETHICS STATEMENT

Written informed consent was obtained from the patients' parents in our study. The purpose of this research was completely explained to the patients' parents and was assured that their information will be kept confidential by the researcher. The present study was approved by the Medical Ethics Committee of the academy.

## CONSENT

Written informed consent was obtained from the patient to publish this report in accordance with the journal's patient consent policy.

## Data Availability

All data regarding this case has been reported in the manuscript. Please contact the corresponding author if you are interested in any further information.
